# Genetic Testing Among Medicaid-Insured Children With Autism and Intellectual Disability

**DOI:** 10.1001/jamanetworkopen.2025.33518

**Published:** 2025-09-19

**Authors:** Tashalee R. Brown, Wei-Lin Lee, Jonas Ventimiglia, Scott D. Grosse, Tess Levy, Audrey Thurm, Julian A. Martinez-Agosto, Lindsay L. Shea

**Affiliations:** 1 Department of Psychiatry and Biobehavioral Sciences, David Geffen School of Medicine at the University of California Los Angeles; 2 Policy and Analytics Center, A.J. Drexel Autism Institute, Drexel University, Philadelphia, Pennsylvania; 3 Department of Pediatrics, University of Minnesota Medical School, Minneapolis; 4 Department of Psychiatry, the Icahn School of Medicine at Mount Sinai, New York, New York; 5 Seaver Autism Center for Research and Treatment at the Icahn School of Medicine at Mount Sinai, New York, New York; 6 Department of Human Genetics, David Geffen School of Medicine at the University of California Los Angeles; 7 Neurodevelopmental and Behavioral Phenotyping Service, Office of the Clinical Director, Intramural Research Program, National Institute of Mental Health

## Abstract

This cohort study examines the frequency of genetic testing among Medicaid-enrolled children with autism and/or intellectual disability.

## Introduction

Autism spectrum disorder (ASD) and intellectual disability (ID) are pediatric conditions that benefit from early diagnosis and treatment,^1^ and professional society guidelines recommend etiological genetic testing upon diagnosis.^2,3^ A genetic diagnosis can enhance medical surveillance and lead to actionable therapies and specialized care.^2,4^ Understanding factors affecting access to genetic testing is essential to ensure these benefits for individuals with ASD and/or ID. This cohort study examines testing frequency among children with ASD and/or ID across demographic factors using Medicaid claims data.

## Methods

This cohort study included participants aged 7 to 17 years in 2019 who were enrolled in Medicaid each year from January 1, 2017, to December 31, 2019. Diagnoses were classified based on established algorithms using the *International Classification of Diseases, Ninth Revision* and *Tenth Revision* diagnostic codes,^5^ which were compared with a random sample of children without ASD and/or ID and participants with epilepsy-only diagnostic codes (eMethods and eTable 1 in Supplement 1). Genetic testing was categorized using Current Procedural Terminology Codes (eTable 2 in Supplement 1). Logistic regression models estimated odds ratios (ORs) and 95% CIs, adjusting for covariates using SAS version 9.4 (SAS Institute). The institutional review board of Drexel University granted a HIPAA waiver. We followed the STROBE reporting guideline. Consent was not required because this was a secondary analysis.

## Results

Our cohort included 885 895 children (mean [SD], 11.82 [3.07] years; 584 327 males [65.96%] and 301 568 females [34.04%]): 388 715 with ASD only, 122 792 with ID only, 82 107 with ASD and ID, 3306 with epilepsy only, and 292 281 without ASD or ID diagnosis codes (Table 1). Receipt of genetic testing during the 3-year study period was 8.44% for children with ASD and ID, followed by 4.80% for ID only and 4.17% for ASD only. The prevalence of genetic testing was lower among children diagnosed with epilepsy (2.44%) and still lower for the general pediatric sample (0.39%). Adjusted odds ratios (AOR) of genetic testing were 10.59 (95% CI, 10.22-11.54) for ASD only, 12.72 (95% CI, 11.94-13.56) for ID only, and 23.17 (95% CI, 21.75-24.69) compared with the random sample. AORs varied to a smaller extent by demographic and geographic factors (Table 1). Females had higher odds than males, and most racial and ethnic groups had lower adjusted odds of genetic testing compared with White children. Children residing in large rural towns had slightly higher odds of genetic testing.

**Table 1.** Patient Characteristics and Adjusted Odds Ratios (aORs) of Genetic Testing

Characteristics	All, AOR (95% CI)^a^^,^^b^	ASD only		ID only		ASD and ID		Patient, No. (%)	
		No. (%)	AOR (95% CI)^c^	No. (%)	AOR (95% CI)^c^	No. (%)	AOR (95% CI)^c^	Random Sample	Epilepsy-only
All (N = 885 895)		388 715 (43.88)	NA	122 792 (13.86)	NA	82 107 (9.27)	NA	292 281 (32.99)	3306 (0.37)
Genetic Testing	NA	16 211 (4.17)	NA	5891 (4.80)	NA	6931 (8.44)	NA	1151 (0.39)	74 (2.24)
Age in 2019, mean (SD)	NA	11.52 (3.05)	NA	12.49 (2.96)	NA	12.58 (3.00)	NA	11.71 (3.08)	11.87 (3.07)
Sex									
Male	1 [Reference]	306 057 (78.74)	1 [Reference]	71 413 (58.16)	1 [Reference]	60 194 (73.31)	1 [Reference]	146 663 (50.18)	1745 (52.78)
Female	1.07 (1.04-1.09)	82 658 (21.26)	1.03 (0.99-1.07)	51 379 (41.84)	1.09 (1.04-1.15)	21 913 (26.69)	1.102 (1.04-1.16)	145 618 (49.82)	1561 (47.22)
Race									
American Indian or Alaska Native	0.69 (0.61-0.78)	4182 (1.08)	0.68 (0.56-0.80)	1286 (1.05)	0.74 (0.56-0.98)	761 (0.93)	0.8 (0.61-1.05)	4227 (1.45)	47 (1.42)
Asian or Pacific Islander	0.66 (0.63-0.68)	9627 (2.48)	0.71 (0.68-0.75)	4130 (3.36)	0.55 (0.51-0.60)	2918 (3.55)	0.68 (0.63-0.73)	12 110 (4.14)	86 (2.60)
Black or African American	0.90 (0.84-0.97)	62 711 (16.13)	1.02 (0.93-1.13)	28 989 (23.61)	0.72 (0.61-0.84)	15 837 (19.29)	0.96 (0.84-1.10)	61 796 (21.14)	784 (23.71)
Hispanic or Latino	0.88 (0.86-0.91)	99 354 (25.56)	0.90 (0.86-0.94)	28 289 (23.04)	0.96 (0.90-1.03)	19 362 (23.58)	0.88 (0.82-0.94)	101 645 (34.78)	959 (29.01)
Multiracial	0.82 (0.72-0.92)	4205 (1.08)	0.85 (0.73-1.00)	883 (0.72)	0.68 (0.48-0.96)	967 (1.18)	0.82 (0.65-1.04)	1562 (0.53)	31 (0.94)
White	1 [Reference]	165 007 (42.45)	1 [Reference]	42 061 (34.25)	1 [Reference]	33 369 (40.64)	1 [Reference]	93 246 (31.90)	1231 (37.24)
Missing or unknown	0.99 (0.95-1.03)	43 629 (11.22)	1.02 (0.97-1.08)	17 154 (13.97)	0.93 (0.86-1.01)	8893 (10.83)	1.018 (0.94-1.10)	17 695 (6.05)	168 (5.08)
Urbanicity									
Urban	1 [Reference]	324 347 (83.44)	1 [Reference]	98 744 (80.42)	1 [Reference]	67 877 (82.67)	1 [Reference]	244 893 (83.79)	2742 (82.94)
Large rural city/town	1.05 (1.01-1.09)	35 782 (9.21)	1.06 (1.01-1.12)	13 886 (11.31)	0.98 (0.90-1.06)	8191 (9.98)	1.09 (1.00-1.18)	26 394 (9.03)	313 (9.47)
Small rural town	1.04 (0.98-1.09)	16 960 (4.36)	0.99 (0.90-1.09)	6308 (5.14)	0.94 (0.84-1.06)	3693 (4.50)	1.10 (0.98-1.23)	12 586 (4.31)	143 (4.33)
Isolated small rural town	0.99 (0.92-1.06)	10 382 (2.67)	0.99 (0.90-1.09)	3418 (2.78)	0.95 (0.81-1.11)	2100 (2.56)	1.01 (0.87-1.18)	7843 (2.68)	NR^d^
Missing or unknown	0.87 (0.70-1.08)	1244 (0.32)	0.86 (0.64-1.16)	436 (0.36)	0.77 (0.47-1.24)	246 (0.30)	0.93 (0.59-1.48)	565 (0.19)	NR^d^

Abbreviations: AOR, adjusted odds ratio; ASD, Autism spectrum disorder; ID, intellectual disability; NR, not reported; NA, not applicable,

^a^ Model adjusted for diagnosis group, sex, race and ethnicity, and urbanicity.

^b^ Includes all children with ASD only, ID only, ASD and ID, and random sample.

^c^ Model adjusted for sex, race and ethnicity, and urbanicity.

^d^ Not reported due to the requirements of the Centers of Medicare & Medicaid Services.

From 2017 to 2019, Medicaid genetic testing claims saw declines in cytogenetics (21.5% to 18.8%), fragile X (16.8% to 15.0%), and microarrays (18.3% to 16.9%). In contrast, gene panel and exome sequencing shares increased from 41.8% to 46.6% and 1.6% to 3.0%, respectively (Table 2).

**Table 2.** Genetic Testing Types by Study Year—All ASD and/or ID Children^a^

Year	Patients, No. (%)				
	Cytogenetics	Fragile X	Microarray	Gene Panel	Exome or genome
2017	7261 (21.53)	5656 (16.77)	6157 (18.26)	14 091 (41.79)	548 (1.63)
2018	5542 (20.39)	4406 (16.21)	4859 (17.87)	11 681 (42.97)	698 (2.57)
2019	4895 (18.83)	3907 (15.03)	4386 (16.88)	12 104 (46.57)	787 (3.03)

Abbreviations: ASD, Autism spectrum disorder; ID, intellectual disability.

^a^ One child may have more than 1 genetic testing record.

## Discussion

During the study period, we found that over 90% of eligible children with ASD only, ID only, and ASD and ID didn’t receive genetic testing. There was only a modestly higher prevalence of testing in individuals with ASD and/or ID compared with individuals with epilepsy only and in the general population. Children with ASD and ID had the highest prevalence of testing, followed by ID only and ASD only. Females and those in large rural cities were more likely to be tested. Most racially and ethnically minoritized groups were less likely to undergo testing compared with White children. Gene panels were the most common genetic test, with slight growth from 2017 to 2019, replacing cytogenetics, fragile X, and microarrays. Exome sequencing nearly doubled but remained low.

These findings revealed gaps in genetic testing use among older Medicaid-insured children and adolescents with ASD or ID and highlight demographic and geographic differences. This corresponded with earlier findings of barriers to clinical genetic testing in specific subpopulations and suggested that clinical practices continue to not align with professional guideline recommendations.^6^

This study has limitations. Medicaid claims data have limited generalizability, lack clinical details, which hinders the establishment of causality between diagnoses and genetic testing, and have gaps in reporting for race and ethnicity. The sample restriction to children age 7 years and older, which was selected because many diagnoses of ID are made after age 6 years, excludes genetic testing that occurred before age 5 years, which may underestimate the prevalence of genetic testing. Our study data are limited to the 2017 through 2019 period, but they provide the most up-to-date reporting available without confounding access barriers driven by the COVID-19 pandemic. Future research should explore health policies and interventions to improve access to genetic testing services among individuals with ASD or ID.

## Notes

Supplement 1.
eMethods.
eTable 1. *International Classification of Diseases, Ninth Revision* (*ICD-9*) and *Tenth Revision* (*ICD-10*) diagnosis codes eTable 2. Genetic testing CPT codes
eReferences.

Supplement 2.
Data Sharing Statement
