# Supplementary material for: Genetic Testing Among Medicaid-Insured Children With Autism and Intellectual Disability
Source: JAMA Netw Open. 2025 Sep 19;8(9):e2533518. doi: 10.1001/jamanetworkopen.2025.33518 (PMC12449721; doi:10.1001/jamanetworkopen.2025.33518)
Supplement: Supplement 1. — eMethods. eTable 1. International Classification of Diseases, Ninth Revision (ICD-9) and Tenth Revision (ICD-10) diagnosis codes eTable 2. Genetic testing CPT codes eReferences. [file jamanetwopen-e2533518-s001.pdf]

Supplemental Online Content

Brown TR, Lee WL, Ventimiglia J, et al. Genetic testing among Medicaid-Insured children with autism and intellectual disability. *JAMA Netw Open*. 2025;8(9):e2533518. doi:10.1001/jamanetworkopen.2025.33518

eMethods.
eTable 1. *International Classification of Diseases, Ninth Revision* (*ICD-9*) and *Tenth Revision* (*ICD-10*) diagnosis codes
eTable 2. Genetic testing CPT codes
eReferences.

This supplemental material has been provided by the authors to give readers additional information about their work.

© 2025 Brown TR et al. *JAMA Network Open*.

eMethods

We utilized longitudinal administrative data from the Transformed Medicaid Statistical Information System (T-MSIS) Analytic Files (TAF) database, covering the period from January 1, 2017, to December 31, 2019, from the Centers for Medicare and Medicaid Services (CMS). The analysis was completed from June 18, 2024, to June 27, 2024. This dataset includes information on enrollment, diagnosis codes, inpatient services, outpatient services, and outpatient drug claims. Individual demographic details are contained in personal summary files. Individuals without information on race, ethnicity, or urbanicity were classified as missing. Race and ethnicity were optionally reported by Medicaid and CHIP beneficiaries (i.e., parent reports) and collected at the state level using a variety of classifications differing in the names and numbers of categories as well as how those categories were combined to create aggregated race/ethnicity categories that conformed to OMB guidelines. States reported to CMS using their own classifications, which were recategorized by CMS under the following combined race and ethnicity categories[1]: American Indian and Alaska Native; Asian, Black, Hawaiian/Pacific Islander; Hispanic; Multiracial; White; or other, which includes non-Hispanic with missing race and missing both race and ethnicity. The race and ethnicity represent the original database item. The study population consisted of children aged 7–17 in 2019 who had complete data throughout the study period and were enrolled in Medicaid for at least 9 months each calendar year from 2017 to 2019. Children with any third-party liability insurance coverage, including private insurance coverage outside of Medicaid, whether primary or secondary, during the study period were excluded. "ASD-only" refers to having at least one inpatient claim or two non-drug claims of any service type linked to diagnostic codes for ASD (eTable 1), without the presence of co-occurring ID. "ID-only" refers to having at least one inpatient claim or two non-drug claims of any service type linked to diagnostic codes for ID (eTable 1), without the presence of co-occurring ASD. "Epilepsy-only", refers to the presence of Epilepsy diagnostic codes without the presence of co-occurring ASD or ID. Diagnoses are based on established algorithms from the Chronic Conditions Data Warehouse[2]. This study includes a randomly selected comparison cohort of Medicaid-enrolled children without ASD or ID ICD-9/10 diagnosis codes who met the inclusion criteria of 9 months of enrollment each study year. Epilepsy-only was included because it is not a neurodevelopmental diagnosis, and it has its own guidelines for genetic testing to act as a comparison to

© 2025 Brown TR et al. *JAMA Network Open*.

the genetic testing frequency in ASD and ID. The primary outcome was genetic testing evaluated the presence of Current Procedural Terminology (CPT) Codes (eTable 2). No adjustments were made for multiple comparisons. Additional details regarding study methods are available as previously published[3].

© 2025 Brown TR et al. *JAMA Network Open*.

eTable 1. ***International Classification of Diseases, Ninth Revision*** **(*ICD-9*) and *Tenth Revision* (*ICD-10*) Diagnosis Codes**

Diagnosis	Reference Period (years)	ICD-9 Codes	ICD-10 Codes	Type of Algorithm	Years of data used	Continuous enrollment requirements (months)[a]	Age range in years in 2016
Autism Spectrum Disorders	2	299.0, 299.00, 299.01, 299.1, 299.11, 299.8, 299.80, 299.81, 299.9, 299.90, 299.91	F84.0, F84.3, F84.5, F84.8, F84.9	At least 1 inpatient claim OR 2 other non-drug claims of any service type with DX codes (any diagnosis on the claim)	2017 to 2019	9	7 - 17
Intellectual Disabilities and Related Conditions	2	317, 318, 318.0, 318.1, 318.2, 319,	F70, F71, F72, F73, F78, F79	At least 1 inpatient claim OR 2 other non-drug claims of any service type with DX codes (any diagnosis on the claim)	2017 to 2019	9	7 - 17
Epilepsy	2	DX 345, 345.0, 345.00, 345.01, 345.1, 345.10, 345.11, 345.2, 345.3, 345.4, 345.40, 345.41, 345.5, 345.50, 345.51, 345.6, 345.60, 345.61, 345.7, 345.70, 345.71, 345.8, 345.80, 345.81, 345.9, 345.90, 345.91	DX G40.001, G40.009, G40.011, G40.019, G40.101, G40.109, G40.111, G40.119, G40.201, G40.209, G40.211, G40.219, G40.301, G40.309, G40.311, G40.319, G40.401, G40.409, G40.411, G40.419, G40.42, G40.501, G40.509, G40.801, G40.802, G40.803, G40.804, G40.811, G40.812, G40.813, G40.814, G40.821, G40.822, G40.823, G40.824, G40.833, G40.834, G40.89, G40.901, G40.909, G40.911, G40.919, G40.A01, G40.A09, G40.A11, G40.A19, G40.B01, G40.B09, G40.B11, G40.B19, G40.C01, G40.C09, G40.C11, G40.C19	At least 1 inpatient claim OR 2 other non-drug claims of any service type with DX codes (any diagnosis on the claim)	2017 to 2019	9	7 - 17

[a] 9-month out of 12-month enrollment was required to account for continuity of enrollment by small disruptions of Medicaid enrollment due to administrative processes[4].

© 2025 Brown TR et al. *JAMA Network Open*.

eTable 2. Genetic Testing CPT Codes

CPT Code	Domain
81228;81229	Arrays
81245;81246;81247;81415;81416;81417;81425;81426;81427	Exome / Genome Sequencing
81243;81244;83897;83900;83909;83891	Fragile X
88271;88272;88273;88274;88275	Molecular cytogenetics
83890;83891;83892;83893;83894;83896;83898;83901;83902;83903;83904;83905;83906;83907;83908;83912;83913;83890;83892;83893	Other
81185;81186;81239;81302;81303;81304;81321;81322;81323;81331;81400;81401;81402;81403;81404;81405;81406;81407;81408;81419;81442;81470;81471;81479	Single genes / Panels
88230;88245;88248;88249;88260;88261;88262;88263;88264;88267;88269;88280;88283;88285;88289;88291	Traditional cytogenetics

eReferences

1. Saunders H, Published PC. Medicaid Administrative Data: Challenges with Race, Ethnicity, and Other Demographic Variables. KFF. April 28, 2022. Accessed July 13, 2025. https://www.kff.org/medicaid/issue-brief/medicaid-administrative-data-challenges-with-race-ethnicity-and-other-demographic-variables/

2. Data Dictionaries. Chronic Conditions Data Warehouse. Accessed May 6, 2024. https://www2.ccwdata.org/data-dictionaries

3. Brown TR, Lee WL, Ventimiglia J, et al. Medicaid claims from 2008 to 2016 indicate low rates of genetic testing among children with intellectual disability and autism spectrum disorder. *Genetics in Medicine*. Published online June 18, 2025:101451. doi:10.1016/j.gim.2025.101451

4. Grosse SD, Nichols P, Nyarko K, Maenner M, Danielson ML, Shea L. Heterogeneity in Autism Spectrum Disorder Case-Finding Algorithms in United States Health Administrative Database Analyses. *J Autism Dev Disord*. 2022;52(9):4150-4163. doi:10.1007/s10803-021-05269-1

© 2025 Brown TR et al. *JAMA Network Open*.
