# Supplementary material for: Genetic Testing Among Medicaid-Insured Children With Autism and Intellectual Disability
Source: JAMA Netw Open. 2025 Sep 19;8(9):e2533518. doi: 10.1001/jamanetworkopen.2025.33518 (PMC12449721; doi:10.1001/jamanetworkopen.2025.33518)
Supplement: Supplement 2. — Data Sharing Statement [file jamanetwopen-e2533518-s002.pdf]

Data Sharing Statement

Brown. Genetic Testing Among Medicaid-Insured Children With Autism and Intellectual Disability. *JAMA Netw Open*. Published September 19, 2025. doi:10.1001/jamanetworkopen.2025.33518

Data
Data available: No
